# Current Role of Lipoprotein Apheresis

**DOI:** 10.1007/s11883-019-0787-5

**Published:** 2019-05-01

**Authors:** Gilbert Thompson, Klaus G. Parhofer

**Affiliations:** 1 Department of Metabolic Medicine, Imperial College London, Hammersmith Hospital, Ducane Road, London, W12 0NN UK; 2 Medical Dept. IV – Grosshadern, Ludwig-Maximilians-University Munich, Munich, Germany

**Keywords:** Homozygous familial hypercholesterolaemia, Heterozygous familial hypercholesterolaemia, Lipoprotein(a), Apheresis guidelines

## Abstract

**Purpose of Review:**

Lipoprotein apheresis is a very efficient but time-consuming and expensive method of lowering levels of low-density lipoprotein cholesterol, lipoprotein(a)) and other apoB containing lipoproteins, including triglyceride-rich lipoproteins. First introduced almost 45 years ago, it has long been a therapy of “last resort” for dyslipidaemias that cannot otherwise be managed. In recent years new, very potent lipid-lowering drugs have been developed and the purpose of this review is to define the role of lipoprotein apheresis in the current setting.

**Recent Findings:**

Lipoprotein apheresis still plays an important role in managing patients with homozygous FH and some patients with other forms of hypercholesterolaemia and cardiovascular disease. In particular, patients not achieving treatment goals despite modern lipid-lowering drugs, either because these are not tolerated or the response is insufficient. Recently, lipoprotein(a) has emerged as an important cardiovascular risk factor and lipoprotein apheresis has been used to decrease lipoprotein(a) concentrations in patients with marked elevations and cardiovascular disease. However, there is considerable heterogeneity concerning the recommendations by scientific bodies as to which patient groups should be treated with lipoprotein apheresis.

**Summary:**

Lipoprotein apheresis remains an important tool for the management of patients with severe drug-resistant dyslipidaemias, especially those with homozygous FH.

## Introduction

A detailed description of the history and development of the extracorporeal removal of plasma cholesterol was published in a previous issue of *Current Atherosclerosis Reports* [1]. This introduction provides a brief account of the evolution over the past 50 years of the various procedures now collectively termed lipoprotein apheresis.

The initial stimulus to undertake a radical approach to lowering plasma cholesterol was the intractable nature and severity of the increase in low-density lipoprotein (LDL) cholesterol that characterised homozygous familial hypercholesterolaemia (FH) and resulted all too often in premature death from atherosclerotic cardiovascular disease. The only LDL-lowering drugs available in the 1960s, nicotinic acid and cholestyramine, were ineffective in this situation so Myant [2] and De Gennes et al. [3] resorted to manual plasmapheresis. This lowered plasma cholesterol but was too slow and labour intensive for prolonged use. However, in 1975, Thompson et al. [4••] overcame these drawbacks by using a continuous flow blood cell separator to repetitively undertake unselective plasma exchange in 2 FH homozygotes. Subsequently, Stoffel et al. [5••] introduced selective removal of LDL by using a cell separator to perfuse plasma through an immunoadsorbent column. The latter procedure is still available [6] but has been largely superseded by methods involving perfusion of plasma or whole blood through affinity columns containing either dextran sulphate covalently linked to cellulose beads [7–9] or polyacrylate-coated polyacrylamide beads [10]. Like immunoapheresis, these bind the apolipoprotein B component of LDL and lipoprotein(a) (Lp(a)) and thus remove from the circulation these lipoproteins and their cargo of cholesterol.

A radically different approach to lipoprotein apheresis involves the extracorporeal precipitation of LDL through the addition of heparin to plasma, the so-called HELP system [11]. Precipitation of LDL occurs without addition of cations if the pH is lowered sufficiently, the precipitate being removed by filtration. Another method of removing lipoproteins from plasma is double filtration plasmapheresis (DFPP) [12, 13]. In this procedure, plasma is separated from blood cells by a hollow membrane filter and then perfused through a second filter which selectively retains smaller plasma components like high-density lipoprotein (HDL) and albumin, but discards larger molecular weight components including LDL and Lp(a).

Acute decreases in LDL-cholesterol after each procedure range from 60 to 80%, depending upon the volume of blood or plasma treated. Although it lowers LDL-cholesterol to a similar extent, DFPP removes more HDL cholesterol than other methods. Haemoperfusion systems are the easiest to use but, like dextran sulphate–based plasma adsorption methods, employ disposable columns and are therefore more expensive than immunoadsorption, which utilises re-usable columns. Dextran sulphate–based methods are probably the most popular and are remarkably safe [14]. A comparison in FH homozygotes of dextran sulphate adsorption and HELP apheresis in Canada showed that the former lowered LDL-cholesterol to a greater extent than the latter (70.5% vs 63%, *P* = 0.02) mainly because it enables a greater volume of plasma to be treated [15].

A recent international survey of the management of FH found that lipoprotein apheresis was available in approximately 60% of 63 countries worldwide [16], cost being a limiting factor. In Germany, where lipoprotein apheresis is reimbursed by the health care system, almost 1300 patients received this treatment at 68 centres between 2012 and 2015 [17]. The clinical indications, current guidelines and evidence base for the efficacy of lipoprotein apheresis in the treatment of patients with severe hyperlipidaemia are discussed in the subsequent sections of this review.

## Current Indications and Recent Guidelines for Lipoprotein Apheresis

Although most lipid guidelines mention lipoprotein apheresis as a therapy of last resort, they differ significantly in defining which patients to treat and under what circumstances [18]. This reflects a lack of convincing outcome trials as most of the evidence supporting the use of lipoprotein apheresis comes from retrospective analyses or extrapolation of intervention studies using lipid-lowering drugs. Since lipoprotein apheresis effectively decreases the plasma concentration of LDL, lipoprotein(a) and triglyceride-rich lipoproteins, it can be hypothesised that lipoprotein apheresis could be used in a number of different clinical settings.

Currently, lipoprotein apheresis is mainly used in two different clinical settings (Table 1):

- Significantly elevated LDL-cholesterol
- Significantly elevated lipoprotein(a)

**Table 1:** Guidelines for using lipoprotein apheresis

Country	Recommendation
USA	• Homozygous FH: LDL-c ≥ 500 mg/dl (12.9 mmol/L) on maximal possible drug therapy • Heterozygous FH: LDL-c ≥ 300 mg/dl (7.8 mmol/L) (0–1 additional risk factor), LDL-c ≥ 200 mg/dl (5.2 mmol/L) (≥ 2 additional risk factors or additional high lipoprotein(a)), LDL ≥ 160 mg/dl (4.1 mmol/L) (if at very high risk)
Germany	• Homozygous FH • Severe hypercholesterolaemia (including but not restricted to heterozygous FH): LDL-c elevated on maximal possible drug therapy (taking the overall risk of the patient into account) • Lipoprotein(a): progressive CVD (clinically and on imaging) despite optimal control of all other risk factors and lipoprotein(a) ≥ 60 mg/dl
Japan	• Homozygous FH • Heterozygous FH: total cholesterol ≥ 250 mg/dl (6.5 mmol/L) on maximal possible drug therapy
UK	• Homozygous FH: LDL-c reduction < 50% on max. drug therapy or LDL-c ≥ 350 mg/dl (9.1 mmol/L) • Other hypercholesterolaemia (including heterozygous FH): CVD progression and LDL-c ≥ 190 mg/dl (4.9 mmol/L) or lower if lipoprotein(a) elevated or LDL-c reduction < 40%
Australia	• Homozygous FH: LDL-c ≥ 270 mg/dl (7.0 mmol/L) on maximal possible drug therapy • Heterozygous FH: CVD and LDL-c ≥ 193 mg/dl (5.0 mmol/L) on maximal possible drug therapy • Alternative criteria (homozygous FH and heterozygous FH): < 50% reduction on maximal possible drug therapy
Spain	• Homozygous FH • Heterozygous FH: LDL-c ≥ 200 mg/dl (5.2 mmol/L) with CVD or ≥ 300 mg/dl (7.8 mmol/L) without CVD

*CVD* cardiovascular disease, *LDL-c* LDL-cholesterol, *FH* familial hypercholesterolaemia

With respect to elevated LDL-cholesterol (LDL-C), there is agreement that patients with homozygous FH inadequately responsive or refractory to lipid-lowering drugs qualify for such treatment [19–25]. Guidelines also agree that in homozygous FH, apheresis therapy should be started as early as possible, preferably in early childhood.

The situation is less clear-cut for patients with heterozygous FH or other forms of hypercholesterolaemia (Table 1). For example, in the USA, apheresis is approved for severe LDL-hypercholesterolaemia which persists despite maximal drug therapy (LDL > 300 mg/dl (7.8 mmol/L) without concomitant cardiovascular disease or > 200 mg/dl (5.2 mmol/L) with concomitant cardiovascular disease) [20]. In Germany, apheresis for elevated LDL-C can be performed if, despite maximal possible drug therapy, LDL-C cannot be reduced sufficiently. No specific threshold is given because the overall risk profile of each patient needs to be considered [22]. In Japan, apheresis is indicated in heterozygous FH if total cholesterol remains above 250 mg/dl (6.5 mmol/L) despite maximal drug therapy [21]. Thus, generally speaking, apheresis can be considered in hypercholesterolaemia other than homozygous FH if atherosclerotic vascular disease is present and progressive and if LDL-C treatment goals, which vary from country to country, are not met despite maximal possible drug therapy (including proprotein convertase subtilisin/kexin type 9 (PCSK9) inhibitors).

Although lipoprotein(a) is a causal risk factor for atherosclerotic disease and although there are only limited means to treat elevated lipoprotein(a) (Lp(a)) levels, the role of lipoprotein apheresis in this context is not well defined. The National Lipid Association and Heart-UK consider elevated Lp(a) as an additional risk factor that should be taken into account when deciding whether lipoprotein apheresis should be used to treat elevated LDL-C [20]. Elevated Lp(a) per se is therefore not an indication. In contrast, in Germany, elevated Lp(a) levels are considered to be an indication for regular apheresis if certain prerequisites are fulfilled, namely if Lp(a) is > 60 mg/dl in patients with progressive cardiovascular disease despite optimal management of all other risk factors including LDL-C [17]. Some of the other guidelines do not mention the role of lipoprotein apheresis for treating patients with elevated Lp(a).

Although lipoprotein apheresis also decreases the concentration of triglyceride-rich lipoproteins, none of the guidelines specify the circumstances under which hypertriglyceridaemia should be treated with lipoprotein apheresis.

## Theoretical and Practical Considerations Governing the Optimum Frequency and Efficacy of Lipoprotein Apheresis Procedures

It has long been accepted that the production rate of LDL, the lipoprotein that transports over 90% of plasma cholesterol in FH homozygotes, obeys zero order kinetics, i.e. it remains constant irrespective of pool size, whereas catabolism of LDL is governed by first-order kinetics, i.e. the fractional catabolic rate (FCR) is constant irrespective of pool size [26]. There is a steep fall in plasma total and LDL-cholesterol immediately after apheresis and then a curvelinear rebound back to the baseline level, the speed of which is largely determined by the FCR of the lipoprotein particle in question. For LDL, this depends upon inherent LDL receptor activity plus the influence of any lipid-lowering drugs on the latter, such as statins.

**Fig. 1:**
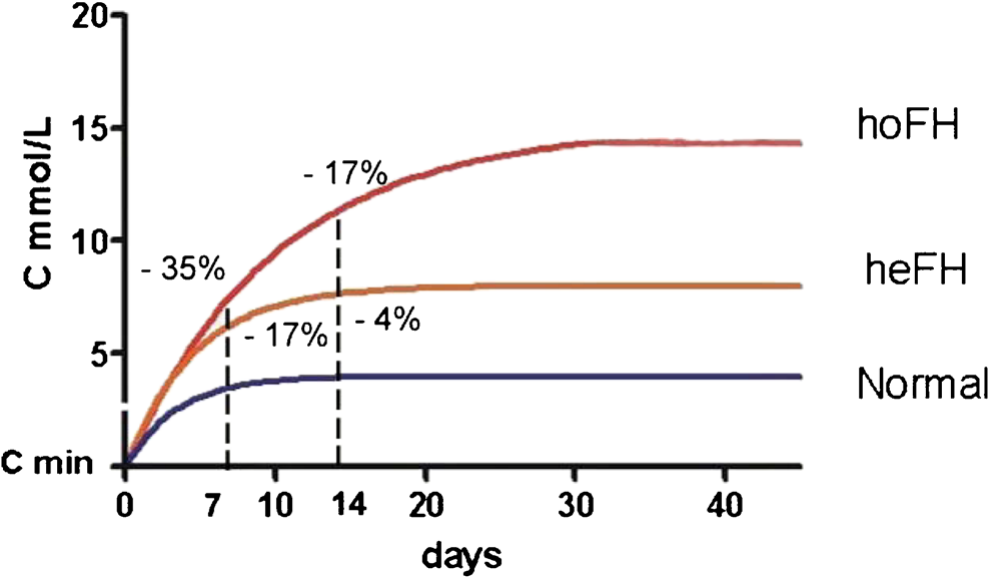
Rebounds in plasma cholesterol (C) from post-apheresis values (C min) are shown in a normal subject, FH heterozygote (heFH) and FH homozygote (hoFH). Values of C expressed as percentages of baseline values in FH patients are shown at 7 and 14 days, illustrating the reductions achieved by weekly versus bi-weekly apheresis respectively

The magnitude of the acute decrease in lipoproteins after apheresis depends upon the volume of plasma treated, treatment of 1.2 plasma volumes (approximately 4 l) resulting in a reduction of 70% below the baseline value. As shown in Fig. 1, the subsequent rebound in plasma cholesterol is fastest in normal subjects and slowest in FH homozygotes, with heterozygotes intermediate. The actual value of total or LDL-cholesterol (Ct) at any given time (t) after apheresis can be calculated from the formula:

$$ \mathrm{Ct}=\mathrm{C}0-\left(\mathrm{C}0-\mathrm{Cmin}\right)\ {\mathrm{e}}^{-\mathrm{kt}} $$

where C0 is the baseline value, Cmin is the post-apheresis value, and k is the FCR [27]. For example, in the homozygote in Fig. 1, if the baseline level of cholesterol of 15 mmol/l is acutely reduced by 70% and the FCR is 0.1, the post-apheresis levels at 1 and 2 weeks will be respectively 35% and 17% below the baseline level. Similarly in the heterozygote, if the baseline level of 7 mmol/l is reduced by 70% and the FCR is 0.2, then post-apheresis levels at 1 and 2 weeks will be 17% and 4% below the baseline level. Hence, apheresis every 2 weeks has a modest cholesterol-lowering effect in homozygotes but virtually none in heterozygotes, in whom weekly apheresis is necessary for any significant effect. This is exemplified by the Familial Hypercholesterolaemia Regression Study, where bi-weekly apheresis (because of operational constraints) plus simvastatin lowered LDL-cholesterol only marginally more in FH heterozygotes than did a bile acid sequestrant plus simvastatin [28].

A similar approach can be used to describe Lp(a) rebound following apheresis. A direct comparison indicates that lipoprotein(a) rebounds at a slower rate than LDL but with a similar monoexponential function [29]. Therefore, if apheresis is performed weekly, Lp(a) concentration will not rebound to its original (pre-first apheresis) value and therefore, a lower level will be achieved.

Plasma levels of LDL and Lp(a) decrease further if apheresis is repeated on a regular basis but stabilise when a new non-steady state is reached. In terms of clinical relevance, the best index of efficacy is probably the interval mean between successive procedures, which can be calculated by integrating the area under the rebound curve or using a modified version of the formula of Kroon et al. [30, 31]. Patients’ compliance is another important factor in determining the long-term benefit of lipoprotein apheresis. A recent survey in France showed an overall compliance rate of nearly 90%, non-compliance being evident mainly in patients undergoing weekly apheresis [32].

## Recent Evidence of Therapeutic Benefit of Lipoprotein Apheresis via Lowering of

- Low-Density Lipoprotein

Data from the German Lipoprotein Apheresis Registry (GLAR), based on over 15,000 apheresis procedures, showed a median acute reduction in LDL-cholesterol of 69% and of Lp(a) of 70% in hyperlipidaemic patients with cardiovascular disease [17]. These reductions were associated with a 97% decrease in the incidence of major adverse coronary events (MACE) during the first year of lipoprotein apheresis compared with the 2 years preceding the start of this treatment. These data were obtained prior to the introduction of PCSK9 inhibitors in Germany.

In the ODYSSEY ESCAPE trial, treatment of FH heterozygotes undergoing lipoprotein apheresis with the PCSK9 inhibitor alirocumab resulted in an additional 54% reduction in LDL-cholesterol. Based on the trial criterion of reducing LDL-cholesterol by ≥ 30% below the baseline value on apheresis, 63% of patients on alirocumab were able to discontinue apheresis altogether and over 90% to halve its frequency [33••]. In contrast, in FH homozygotes on apheresis in the TAUSSIG study, reductions in LDL-cholesterol after the addition of the PCSK9 inhibitor evolocumab averaged 23% [34•]. This was much less than that observed in FH heterozygotes on evolocumab [35] and the decrease in Lp(a) was also less, only 12%.

One of the factors influencing the LDL-lowering response of homozygotes to evolocumab was LDL receptor status. The 10% who were receptor negative showed only a 6% decrease in LDL whereas those who were receptor defective had a 24% decrease. Although PCSK9 inhibitors clearly have considerable potential as an alternative to apheresis in the treatment of patients with statin-refractory heterozygous FH, their usefulness in homozygous FH and in patients with raised Lp(a) levels is less obvious and in most instances, they will complement rather than replace lipoprotein apheresis.

Another adjunctive drug, whose action is independent of LDL receptor status, is the microsomal triglyceride transfer protein (MTP) inhibitor lomitapide, which reduces LDL-cholesterol by 50% in FH homozygotes [36]. Its efficacy is similar irrespective of whether such patients are or are not on lipoprotein apheresis and in about 50% of instances, it reduced their LDL-cholesterol to < 2.5 mmol/l (96 mg/dl) [37]. However, its long-term safety remains under scrutiny. The apoB synthesis inhibitor mipomersen was used in another study in patients undergoing regular apheresis but did not result in a decreased apheresis frequency and was associated with a high incidence of side effects [38].

A frequent cause of morbidity and mortality in homozygous FH is atherosclerosis of the aortic root. The location and severity of atheroma at this site in FH homozygotes, but not in heterozygotes, is identical to that seen in cholesterol-fed rabbits and is attributable to the severity of hypercholesterolaemia in both these situations [39]. A 50-year long survey of UK patients found that the occurrence of aortic stenosis was lower in patients who started treatment during the 1990s as opposed to those treated in the pre-statin era (33% vs 77%, *P* = 0.02), reflecting better control of serum cholesterol by apheresis and statins [40]. A French study of children with homozygous FH showed that the frequency of aortic stenosis and need for surgery were associated with the age at which lipoprotein apheresis was initiated [41]. Those with aortic root atheroma started apheresis at age 10 whereas those without atheroma had started it earlier, at age 5.

Recent evidence that effective lipid-lowering therapy increases life expectancy came from a retrospective survey of 133 homozygotes in South Africa and the UK who were divided into quartiles according to their on-treatment levels of serum cholesterol from 1990 to 2014 [42•]. Patients in quartile 4, with an on-treatment serum cholesterol > 15 mmol/l (584 mg/dl), had a hazard ratio of 11:5 for total mortality compared with those in quartile 1, with an on-treatment cholesterol of < 8 mmol/l (313 mg/dl). Those in quartiles 2 and 3 combined, with an on-treatment cholesterol of 8  (313 mg/dl) –15 mmol/l (584 mg/dl), had a hazard ratio of 3:6 compared with quartile 1. These differences were statistically significant (*P* < 0.001) and remained so after adjustments for confounding factors (*P* = 0.04). Significant differences between quartiles were also evident for cardiovascular deaths and MACE. It is noteworthy that 50% of UK patients were on apheresis versus 13% of South African patients and that 60% of the former but only 19% of the latter were in quartile 1, reflecting the fact that reductions in total cholesterol in patients on apheresis averaged 57% in the UK versus 32% in South Africa (*P* = 0.01). This study provides strong evidence that the extent of reduction of serum cholesterol achieved by a combination of therapeutic measures, including lipoprotein apheresis, statins, ezetimibe, and evolocumab, is a major determinant of survival in homozygous FH. As stated in an accompanying editorial, at long last, there is “light at the end of the tunnel” for homozygous FH [43].

## Recent Evidence of Therapeutic Benefit of Lipoprotein Apheresis via Lowering of

b. Lipoprotein(a)

An elevated Lp(a) level is an independent risk factor for atherosclerosis [44]. Thus, it can be expected that lowering Lp(a) levels translates into clinical benefit. It is however unclear how much Lp(a) must be decreased to achieve significant risk reduction. In a recent study based on genetic data, it was hypothesised that a decrease in Lp(a) concentration of > 100 mg/dl is required to achieve a benefit equivalent to 1 mmol/l (39 mg/dl) of LDL-cholesterol lowering [45]. On the other hand, data from the Odyssey Outcomes trial indicate that much less reduction in Lp(a) was beneficial (1 mg/dl reduction resulted in 0.6% relative risk reduction; thus, about 35 mg/dl lipoprotein(a) reduction would lead to the same risk reduction as 1 mmol/L (39 mg/dl) of LDL-C reduction) [data only published in abstract form]. The topic is further complicated by the fact that so far there are no drugs available that solely decrease Lp(a) concentrations. Niacin decreases Lp(a) but has also multiple other effects on lipoproteins and was not shown to have clinical benefit [46]. PCSK9 inhibitors can also decrease Lp(a) concentrations but also (and primarily) decrease LDL-cholesterol, which makes it difficult to decide how much of the clinical benefit relates to LDL-cholesterol reduction and how much to lipoprotein(a) reduction. Similarly, most lipoprotein apheresis methods decrease both LDL and Lp(a) concentrations, again making it difficult to dissect out the effect of lipoprotein(a) reduction. In addition, there are no adequate clinical endpoint trials evaluating the effect of apheresis in patients with elevated Lp(a).

As discussed earlier, an older trial evaluated whether in patients with CAD and heterozygous FH (*n* = 39) bi-weekly apheresis in combination with simvastatin (40 mg/day) is superior to simvastatin (40 mg/day) in combination with colestipol (20 g/day) [28]. After 2.1 years, there was no significant difference between the two groups and therefore, the authors concluded that “decreasing Lp(a) seems to be unnecessary if LDL-C is reduced to 3.4 mmol/l (132 mg/dl) or less”. However, patients had a low Lp(a) baseline level and only a modest Lp(a) reduction with apheresis (mean reduction 10 mg/dl). In a subsequent angiographic trial, it was evaluated whether a specific Lp(a) apheresis (Lipopac apheresis) plus statin reduces CHD progression compared to statin alone in 30 patients with CHD and elevated lipoprotein(a) (> 50 mg/dl) [47]. After 18 months, apheresis-treated patients showed significantly more regression and less progression. Again, this trial was limited by a small number of subjects and the lack of reporting of clinical events.

Recently, an analysis of the German Lipoprotein Apheresis Registry for the period 2012–2015 was reported and showed acute reductions of LDL-cholesterol and Lp(a) of 68.6% and 70.4% respectively [17]. The data showed a dramatic reduction (− 97%) of cardiovascular events when the period before initiation of apheresis was compared to the period of regular apheresis. This very impressive reduction must be interpreted with caution as the setting is not randomised or controlled. Another publication showed significant reduction of interventions in patients with peripheral artery disease after initiation of apheresis (observational data) [48].

This recent analysis confirms previous German evaluations and also a study from Italy evaluating cardiovascular events before initiation of apheresis and during regular apheresis therapy [49, 50•, 51, 52]. In two of the German studies, only subjects with isolated Lp(a) elevation were included (with LDL < 2.5 mmol/l (97 mg/dl) on statin therapy) [51, 52], while in the third study, patients with concomitantly elevated LDL-cholesterol were also included [50•]. The event rate decreased in all 4 studies dramatically after initiation of regular apheresis but these observations are severely limited by the lack of a control group. Progression of disease and recurrent events are the main reasons for starting a patient on apheresis. Thus, it is not surprising to observe a very high event rate in the time period before regular apheresis. As outlined elsewhere, it is impossible to confirm the true effect of apheresis without an adequate control group [53, 54].

## Recent Evidence of Therapeutic Benefit of Lipoprotein Apheresis via Lowering of

c. Triglyceride-Rich Lipoproteins

Severe hypertriglyceridaemia (> 10 mmol/l; ca 900 mg/dl) due to increased levels of very low-density lipoprotein (VLDL), chylomicrons and remnant particles is a recognised cause of acute pancreatitis. In these circumstances, plasma exchange with a centrifugal cell separator enables triglyceride levels to be drastically reduced with a rapid resolution of abdominal pain [55]. Hitherto there has been no evidence that this approach reduces morbidity and mortality [56] but recently, Chang et al. [57] showed that in patients with extreme hypertriglyceridaemia (> 56 mmol/l; ca 4900 mg/dl) and acute pancreatitis, treatment with DFPP halved the duration of hospitalisation compared with patients receiving conventional therapy. However, it remains uncertain whether apheresis can reduce mortality in this situation [58].

## Future Prospects for Lipoprotein Apheresis in the Light of Recent Advances in Lipid-Lowering Drugs

Lipoprotein apheresis is not only a modality that has enabled patients with severe hypercholesterolaemia and elevated lipoprotein(a) to be treated but it has also been used as a tool for the better understanding of the regulatory processes involved in lipoprotein metabolism and has thereby advanced knowledge [29, 59–62].

In recent years, new drugs have been brought to the market that effectively treat many patients with severe hypercholesterolemia without resorting to apheresis. The availability of PCSK9 inhibitors decreases the necessity for apheresis dramatically as most patients with heterozygous FH and other forms of hypercholesterolaemia respond very well to this therapy [33••]. From the “LDL-perspective”, only patients with homozygous FH and a limited number of patients with severe forms of heterozygous FH or patients intolerant to any form of lipid-lowering drugs remain potential candidates. With further drugs such as ANGPTL3 inhibitors and bempedoic acid being developed, this group may decrease further [63].

Similarly, potent drugs are being developed for decreasing Lp(a). Of particular interest is an antisense oligo nucleotide that can decrease Lp(a) by more than 70%, which is much greater than the observed interval mean reduction during regular apheresis [64•]. Assuming safety, it can be anticipated that these drugs will have a similar effect on apheresis for elevated Lp(a) as had PCSK9 inhibitors on apheresis for elevated LDL-cholesterol and they will eventually further decrease the number of patients requiring apheresis.

However, we should keep one thing in mind: patients treated by regular apheresis have the advantage of being seen by the same medical team on a very regular (weekly or biweekly) basis. This tight control and guidance improves compliance (generally speaking) and allows medical issues to be discussed regularly in a familiar setting. Although this effect is hard to quantify, it would be surprising if it did not also affect the cardiovascular event rate. Obviously, drug therapy gives the patient more “freedom” but maybe at the cost of less strict medical surveillance.

## Conclusions

Even after recent dramatic improvements in drugs affecting lipid metabolism, lipoprotein apheresis still has its role in treating patients with certain dyslipidaemias. While most patients with heterozygous FH or other forms of elevated LDL-cholesterol can now be treated with drugs, apheresis remains a therapy of last resort in those not responding or intolerant to drugs and is still the gold standard for patients with homozygous FH. It is not only very efficient in decreasing LDL-cholesterol but also very safe and, unlike lomitapide, it can be used in children. In addition to its role in treating severe forms of LDL-hypercholesterolaemia, it is also used in patients with severe elevations of Lp(a) and atherosclerotic disease, although its role in this situation is less well defined. While the number of patients requiring apheresis will probably decrease as new drugs are developed, it will remain a therapy to be kept in reserve for certain types of patient.
